# Long-term couple relationships - stress, problems and coping processes in couple counseling: Insights based on five case studies with five long-term couples

**DOI:** 10.3389/fpsyg.2022.866580

**Published:** 2022-10-20

**Authors:** Ute Kieslich, Gisela Steins

**Affiliations:** ^1^Faculty of Educational Sciences, University of Duisburg-Essen, Duisburg, Germany,; ^2^Institute of Psychology, University of Duisburg-Essen, Duisburg, Germany

**Keywords:** couple counseling, long-term couple relationships, coping processes, problem areas, stress factors

## Abstract

In the course of demographic change, the proportion of older people in many countries is rising continuously and more and more people are experiencing a long time together as a couple. In old age, subjective wellbeing and health aspects are associated with partners’ satisfaction with couple relationship. The need for couple counseling in old age is growing in parallel with demographic developments. However, empirical studies on couple therapy with older people in long-term couple relationships exist to date only to a limited extent. The present contribution deals with this knowledge gap. In an explanative two phases design, research has been conducted with long-term couples in couple counseling of which we would like to present here the central qualitative study. The aim is to be able to describe details of these factors. Older couples who have lived in long-term couple relationships were interviewed after using standardized questionnaires concerning the individual and couple-related stress factors and problems at the beginning (*N* = 62) and the end (*N* = 36) of their couple counseling process. Five couples from this study were the interviewees for the following study. The couples were interviewed separately. In this interview study and central part of this contribution, the stress factors, problem areas and coping processes of the older couples were examined. The results serve as a suggestion for further research and can only be interpreted with caution due to the small sample of five case studies: The central results of the study are summarized in a circular process model and are discussed in the light of relevant theoretical approaches. The culmination of massive chronic and acute strains and losses associated with feelings of excessive demands and desperation gave rise to emotional alienation of the partners. In the course of couple therapy, the partners mutually opened their thoughts and emotions and modified their previous dysfunctional pattern of interaction. Thus, emotional and physical rapprochement of the partners was fostered with the effect that subjective load of the partners and dissatisfaction of the couple relationship decreased, and subjective emotional wellbeing increased. To prevent negative emotions and destructive conflicts with their spouse, some of the interviewed partners actively used avoidance strategies in couple interactions. Upon completion of couple therapy changes in the couple relationships appeared instable as soon as overcharging stress factors occurred again. The results suggest that an approach to couple therapy for older people in long-term couple relationships should prioritize emotional safeness and attachment in couple relationship to facilitate constructive conflict management. The couple therapy process should emphasize emotion regulation strategies based on age-related strengths and consider age-related vulnerabilities. Moreover, long-term couples may benefit from catamnestic consultation appointments to consolidate the developed changes.

## Introduction

In Germany, as in other countries, the increasing life expectancy means that more and more people are spending a long time together as couples. In its report, the German Centre for the Aging (DZA) gives development trends on the lifestyles and couple relationships of older people (Nowossadeck and Engstler, 2013). Due to increased life expectancy, a longer cohabitation of couples can be observed among those aged between 70 and 85. The post-parental phase, i.e., the time spent together as a couple after the children have grown up, has now become the longest phase in the life course. The need for couple counseling in old age is growing simultaneously with the demographic development (Ehe-, Familien- und Lebensberatung im Bistum Münster, 2022). The proportion of clients who are older than 60 years is up to 15% in institutional counseling centers under Catholic sponsorship. Empirical studies on couple therapy with older people in long-term couple relationships, however, only exist to a limited extent. The study presented here deals with the phenomena of older age, individual stress factors, problem areas in long-term couple relationships and corresponding coping processes in the context of couple counseling. With this study, we intend to gain a deeper understanding of the dynamics of coping processes through a detailed insight into the perceived problems of ten people from five couples. In the following, we elaborate on the requirements and the theoretical background for our research.

Interventions that have proven successful in couples counseling with younger couples sometimes turn out to be less effective in counseling with long-term couples. In older age, the transition to retirement is a critical transition phase requiring to reorganize distance and closeness as a couple. Riehl-Emde (2016a) names further essential development tasks: increasing emotional dependence on each other, facing age-related limitations and experiences of loss, mutual care and shared stimulating experiences. These phenomena require the counseling approaches and couple therapy interventions to be tailored for this target group and developed accordingly (Riehl-Emde, 2016b).

### Theoretical background

Predictors of psychological wellbeing and life satisfaction in old age are the subjective state of health, namely, the presence of a partner with whom there is a bonding relationship, and satisfaction with one’s own social relationships (Martin et al., 2000). In view of declining problems like physical strength, health restrictions, psychological stress and withdrawal from professional roles, the couple relationship is important as a stabilizing factor in old age. On the one hand, since the relationship with a partner is the most important attachment relationship in adulthood, a long history as a couple should be seen as a resource. On the other hand, the transition to the old age phase brings new challenges for the individual and the couple system which are associated with very specific development tasks. In older age, critical life events or human borderline situations are more likely to occur (Staudinger and Dittmann-Kohli, 1994). Among other things, couples aged 55 and older are faced with the task of coping with various physical and psychological stress factors and limitations as well as role changes in the private and professional spheres. In the sense of self-organization processes in social systems (Haken and Schiepek, 2006; Schiersmann and Thiel, 2012), long-standing couples in old age are challenged to form qualitatively new organizational structures in their couple system (*cf.* Theory of self-referential systems, Luhmann, 2021).

The approach of lifespan developmental psychology (Brandtstädter and Lindenberger, 2007) offers a suitable theoretical framework for looking at the self-regulatory processes and abilities of individuals as well as dyadic processes in shaping long-term couple relationships in older age. Mainly relevant here are the theoretical approaches and research findings on action motivation and emotion regulation in older people in long-term couple relationships (Carstensen and Lang, 2007; Charles and Hong, 2016; Carstensen, 2021).

Emotional goals become more important in older age in connection with the maintenance of subjective wellbeing. Familiar and meaningful relationships such as the couple relationship are preferred and less meaningful social contacts are reduced (Carstensen and Charles, 1998). Recent models of socio-emotional selectivity theory address age differences in specific areas of emotional and cognitive functioning by combining the developmental domains of meaning and emotion. Here, socio-emotional strengths of older people, for example, emotion regulation competencies and emotional ambiguity tolerance, which are particularly evident in couple and close family relationships are addressed. At the same time, these models take into account the vulnerabilities of older people, which become apparent when stress and emotional arousal persist. In such situations, older people can only downregulate their arousal with a time delay due to age-related changes in physiological processes (Charles, 2010; Charles and Luong, 2013). In order to avoid negative affect and escalation on the couple level, and to maintain subjective wellbeing, older people use different interactional and problem-solving strategies in close relationships than younger people (Holley et al., 2013).

Integrative theoretical models that address the intersection of couple relationships, emotions and health provide a constructive framework for studies with long-term couples (Christensen et al., 2006, 2015). Corresponding research findings show that the interaction and relationship processes of couples are linked to the biological mechanisms in the partners, which are in constant alignment. Both components in turn, affect the individual health of the partners (Ryff et al., 2001; Uchino, 2013). These reciprocal or synchronous patterns in couple relationships have a clear impact on the health status of the partners (Butler, 2011). In stressful life situations, both mutual supportive behavior and tensions in the long-term couple relationship can be understood as the results of such complex interpersonal patterns of emotion regulation.

The approach of lifespan developmental psychology also offers a suitable theoretical framework for reflecting on the processes of couple counseling with older people in long- term couple relationships. Older people are characterized as active shapers of their life span and their relevant relationships, especially when facing age-related limitations and experiences of loss.

### The present research

Little is known about the specifics of couple design and problem solving in the context of couple therapy processes in the life phase of old age. The research presented here aims to help fill this gap. The focus of our research is predominantly on the self-regulatory abilities of individuals with regard to various stresses as well as dyadic processes in the shaping of long- term couple relationships in old age. The research is explorative in character, focuses on the content of the couple relationship and consistently focuses on the subjective perspective of people aged 55 and older who live in long-term couple relationships and have received couple counseling. The structuring aspects of the study are the stress factors, problem areas and coping processes of the study’s participants.

Following a dyadic approach, the present study is particularly interested in the couple interaction patterns that emerge in the assessment, coping and adaptation to various physical and psychological stress factors and limitations as well as role changes in the private and professional sphere in the context of couples counseling. With this study, we intend to gain a deeper understanding of the dynamics of coping processes through a detailed insight into the perceived problems of ten people from five couples. Through interviews, we try to learn more details about problem-solving dynamics through subjective approaches. Although we know from international research about some problem areas that are more likely to occur (such as experiences of loss) and we know about changes in emotion regulation strategies with increasing age (see Theoretical part), we do not know so much about the dynamics of problem solving. It may also be necessary to strongly contextualize the approach to the issue; it is likely that problems and problem-solving dynamics vary with cultural, economic and other factors. Therefore, the aim of the study presented here is a very limited and small one, namely to start *via* the intensive and systematic processing of interviews with a very selective sample. We think the results are interesting for all researchers who deal with coping strategies of long-term couples and also therapists who have long-term couples as clients will certainly benefit from the results for the design of the therapeutic process.

Key results of a previous questionnaire study with long-term couples show that over the period of couples counseling, the subjective health complaints of the individual persons reduced and the overall burden on the couple relationship decreased. However, dissatisfaction with affective communication and emotional intimacy in the relationship with the partner as well as dissatisfaction with the areas of aggression and problem solving in the couple relationship had increased from the beginning to the end of the couples’ consultation.

The subsequent qualitative study presented here has a predominantly exploratory character and is intended to provide an insight in stress factors, problem areas and coping processes in the context of counseling with older people in long-term couple relationships. This is not a purely evaluative procedure with regard to the results of counseling. The focus of the study is on content aspects that move couples in long-term relationships to seek out couple counseling. The questions and topics from the previous questionnaire study are to be deepened (Mayring, 2001).

The study includes qualitative individual interviews with a small sample of couples aged 55 and older. The study focuses on the clients’ subjective experiences and assessments of their individual stresses and strains and the quality of their couple relationship during the period of couple counseling. The reasons for couple counselling, the concerns and expectations of the partners regarding the couple counselling process, their individual burdens, their assessment of the quality of their couple relationship, the problem and conflict areas and the development and coping processes at the individual and couple level.

## Materials and methods

From the proven pool of methods used in qualitative research, individual interviews were conducted with guiding questions in the background, which are open and narrative (Mey and Mruck, 2011; Kruse, 2015). Qualitative interviews are suitable for capturing the inner view of the interviewees on an area of experience and getting to know their thoughts, feelings, evaluations and intentions in a closer and more differentiated way (Patton, 2015, p. 423 ff.). In this study, our interest is focused on those seeking advice in long-term relationships as actors in the couple counseling process. There were three different couple counselors in five couple counseling processes. Three processes were carried out by the same couple counsellor. The study uses 10 qualitative individual interviews with 10 partners from five long-term couples to examine the subjective perspective in the context of couple counseling. The study comprises 10 participants from the pool of the antecedent questionnaire study, so we have a base of five case studies for our analysis.

Hildenbrand cites aspects of a “theory of professional action in counseling and therapy” (Hildenbrand, 2008, p.38), which are based on the Grounded Theory research style (Strauss, 1991). The central elements of this theory are taking the perspective of the person seeking advice and then to use it as a starting point in order to gain new aspects for the processes in the counseling context and in the real life of the person seeking advice through asking questions. Through the active participation of those seeking advice, data are generated which enable a true-to-life, concrete picture of their perceptions, interpretations and processes of change in the period of their couple counseling.

The following specific questions arise: What is the subjective view of the clients on their personal situation and their couple relationship at the start of couple counseling? What was the reason to ask for counseling at this particular time? In retrospect, what changes do the interviewees perceive over the period of the couple counseling, in relation to themselves, their partner and the quality of the couple relationship? How do they assess the quality of their couple relationship during the period of couple counseling and after the counseling process has ended? What else was important to the clients during the couple counseling process with regard to themselves and their partners? From the subjective point of view of the interviewees, what led to probable changes in the relationship with the partner and in the patterns of interaction as a couple? Sexual (dis)satisfaction in the couples’ relationships, the quality of time spent together and the relationships between generations can be relevant topics in the interviews.

### The interviewer

The interviewer was 53 years old at the time of the interviews and is herself a psychologist in couple counseling. The interviewer had direct background information regarding the interviewed clients in six of the 10 interview situations as she had completed a couple counseling process with them as well. The relationship based on trust with these interviewees already existed due to the previous joint counseling process and did not have to be established at the beginning of the interview. On the other hand, respondents may have censored their observations on the counseling process and the relationship with the counsellor in terms of social desirability if the interviewer was also the former counsellor (Crane et al., 2002). The interviewer’s experience during the interviews showed that the interviewees tended to treat the interviewer as a neutral authority.

### Sample

The sample studied consisted of *N* = 10 clients, five wives and five husbands in five different heterosexual relationships. All 10 persons interviewed had received counseling in the couple setting within the framework of marriage, family and life counseling in various cities in Germany. They were between 55 and 72 years old at the time of the interview. The couples were all in their first marriage and the duration of the marriage was between 24 and 48 years. All couples have adult children aged between 20 and 45 years. Four of the couples had received counseling in a couples’ setting, one couple had first participated in a couples’ group and then received counseling as a couple. All the couples volunteered to participate in the study after asking for information *via* the various counseling centers.

### Conducting the interviews

The 10 interviews were held in the premises of the respective counseling center where the clients had previously received couple counseling. The environment was therefore familiar to the people interviewed. The interviews were conducted individually with the partners. A partially standardized procedure was chosen for the interviews. The interview questions were formulated largely openly in accordance with the grounded theory approach (Strübing, 2004). With the exception of the opening and closing questions, the order of the questions was not fixed and was flexibly adapted to the course of the survey in order not to interrupt the flow of the interviewees’ speech (Mayring, 2002, p. 66). The interviews were designed in the continuum between structuring and openness in the direction of narrative interviews with guiding questions in the background. The agreement to the audio recording of the interview and notes by the interviewer was clarified in each case. Consent to the anonymized use of the interview content within the framework of the scientific investigation was obtained in writing (primary and secondary use of the audio data). The approximate duration of the interview was announced.

The length of the interview was determined on the one hand by the narrative flow of the interviewee and on the other hand by the demands of the interviewer, which resulted from the interview guidelines. The interviews lasted 31 min in the shortest interview and 88 min in the longest. The average duration was 49.5 min.

### The interview

The narrative interview type was chosen, modified by a demand orientation (Helfferich, 2011, p.16). The interview guideline is not very structured and begins with a rather broad narrative challenge. The demands listed below were introduced when the interviewees did not spontaneously comment on the topic complexes, whereby the immanent demands listed in the guide were used among others.

In doing so, the narrative of the interviewees was poorly controlled. Introduction: “I would like to talk to you about your very personal experiences during the period of couple counseling in the marriage, family and life counseling center; Request for a narrative:” You have registered for counseling together with your wife / husband. How did it come about, please tell us!”; Immanent requests: “What were the problems about?,” “Can you tell us about the situation in more detail?,” “Is there an explanation?”

Immanent enquiries: “What concerns and expectations brought you to the counseling session with?,” “What has happened between you and your husband/wife during the counseling period?,” “What is your personal impression of your development as a couple during this period?,” “Were there turning points, outstanding events, changes?,” “I notice that you did not mention whether the events and changes in your couple’s relationship were linked to your experiences in couple counseling. Can you say anything more about this?,” “What did you use the counseling for and how did you use it?,” “What was beneficial or detrimental to you during the counseling process?”; Balancing: “Which of the things you have described was particularly important for you?”

#### Visualization parallel to the interview

**Figure 1:**
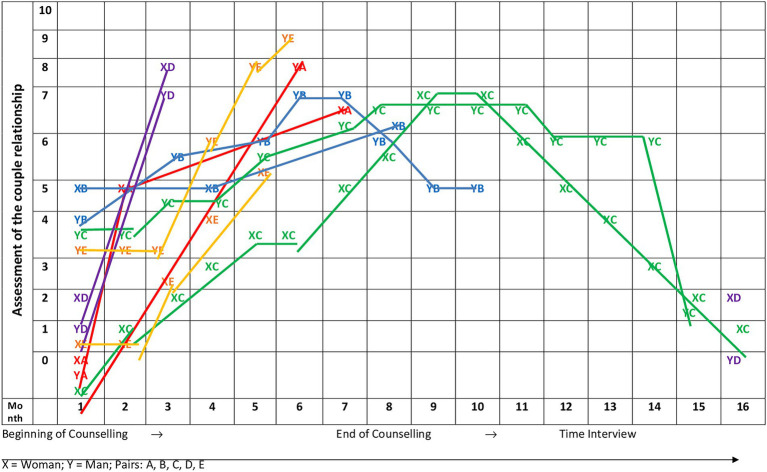
Satisfaction with the couple’s relationship step by step. From the beginning of the counseling session, over the course of time until the end of the counseling session.

During the course of the interview, the interviewees were asked to mark their own satisfaction with the couple’s relationship step by step (Figure 1) at the beginning of the counseling session and over the course of time until the end of the counseling session. The X-axis represents the times of the counseling process, the Y-axis offers a scale from 0 (lowest satisfaction with relationship) to 10 (highest satisfaction with relationship), on which the subjectively perceived quality of the couple relationship is to be assessed. During the interview, the timeline was repeatedly referred to and the respondents were asked for their retrospective assessment of the subjective quality of the couple’s relationship at the respective time mark. The interviewees themselves marked the blank coordinate template with crosses. We then connected these crosses with lines for a course. Some crosses were made on the line, some in between. We tried to include this in the picture in order to clarify the heterogeneity of the subjective perspectives (Figure 1).

### An example of a case study

At this point we list a summary description of a case study so that it becomes very clear what individual wealth of detail already exists in the problem of a single couple. We have chosen couple E for this presentation.

Case study: Couple E. “He was talking to the dog before. And now he talks to me.”

Mr. E. was 59 years old at the time of the interview, Mrs. E. 60. They had been married for 37 years and had three adult children aged 36, 32 and 28. The youngest daughter moved out during the year in which the couple counseling took place. Contact with the two older adult children was described as good by both. The youngest daughter was at times involved in her parents’ conflicts.

Mr. E. used to work full time in the accounting department of a company and was retired at the time of the couple counseling. Mr. E. had been an authorized accountant in a company for a long period of time. He enjoyed the trust of his boss and his colleagues, was committed to a considerable extent in his field of work and helped to build up the company. After the company was taken over by another owner, he could not find an equivalent position in the new system, which hurt him very much and at the same time triggered existential fears in him. In connection with these stresses and legal disputes about his job, he developed a depressive illness. Due to massive physical complaints–several herniated disks–he was granted a severe disability which enabled him to leave his job prematurely. Mr. E. had been in psychotherapeutic treatment for depression for several years before the couples counseling process began. Almost at the same time as the youngest daughter moved out, another personal experience of loss occurred. The dog that had lived in the family for a long time had to be put down. Mr. E. had developed a special relationship with the dog, as it was a helpful resource for him during the phase of his depressive symptoms.

Mrs. E. was employed part-time in the administrative area of a hospital ward. She also suffered from various health restrictions. Approximately 5 years before the couples counseling started, she had been suffering from depression. She had made use of psychological psychotherapy and had good experiences. As a result of a gynecological operation, Mrs. E. experienced chronic problems and pain during sexual intercourse, which led her to avoid intimate contact with her husband. In the year prior to the couples counseling process, she suffered a double-sided hearing loss, which caused her to have chronic hearing loss. Due to her hearing loss, Mrs. E. had great problems at her workplace.

She was also burdened as a carer for her mother, who was in need of care due to dementia. Attempts to care for her mother in a home environment failed. Therefore, Ms. E. had to organize a place for her in a nursing home. With her income, she herself was not able to cover the costs. Mrs. E. was burdened by the prospect of having to draw on the joint savings that her husband and she had set aside in the near future to finance the care place for her mother.

At the time of registration at the counseling center, couple E. had an accumulation of stresses due to physical and mental illnesses. Their own individual and couple-related coping options were exhausted. Their roles had changed due to Mr. E.’s early retirement due to illness so that the regulation of distance and closeness had to be done anew. As a result of the negative stress, Mrs. and Mr. E. increasingly withdrew from emotional contact with each other and thoughts of separation arose. Their sexual relationship was disturbed. Several experiences of loss occurred in quick succession. Both saw the newly developed open communication about contentious issues and the design of joint activities as decisive steps in the couple counseling process. Mr. E. emphasized his change of perspective with regard to his wife and her life achievements as his personal relevant change. By consciously turning toward her, Mr. E. was able to get closer to his wife again. He was able to recognize and appreciate the support he had received from his wife and children during the crisis.

Especially the suggestion in the couple counseling sessions to talk openly about problematic issues in the couple relationship seemed threatening to him at first. He feared that quarrels between him and his wife at home might increase, without any positive effect. Mr. E. experienced a turning point in this regard in the further course of the couple counseling. He had the impression that he and his wife had found a new way of dealing with difficult situations within the couple relationship.

For Mrs. E. it was significant that her husband was now genuinely interested in her and understood her perspective. He had opened up emotionally to her in the couple sessions and talked about his thoughts and feelings. In turn, Mrs. E. changed her interaction strategy in potential conflict situations by not pursuing long-known disagreements and conflictual differences between herself and her husband and instead reacting with composure. She did not address certain aspects in various situations, which made her feel “looser” herself. What was not addressed during the couple counseling sessions was shared sexuality. Mrs. E. brought this topic up on her own in her individual interview.

The counseling took place exclusively in a couple setting over eight sessions, each lasting 90 min. The counseling sessions took place at intervals of about 2 weeks and the counseling process lasted 4 months. The interviews took place 2 weeks after the end of the couple counseling, one after the other, on 1 day at the counseling center.

### Qualitative content analysis

The approach to the interview texts was based on the qualitative content analysis. The aim is to analyze the interview statements of the interviewees, which were fixed *via* transcripts, and to draw conclusions in order to answer the research questions. In this way, the complex material is filtered and structured with regard to the aspects relevant to this study. Due to the exploratory nature of the study, we are most likely to refer to the content- structuring variant, the aim of which is to record topics in the present material (Kuckartz, 2012). Deductive and inductive steps are often combined (Kuckartz, 2007; Schreier, 2014). Deductively derived from the research literature and theoretical models, categories that relate to potential individual and couple-related stress factors and problem areas in old age emerge, such as chronic diseases or normative crises like the departure of adult children and the beginning of retirement.

Patton refers to the approach of “grounded theory” (Glaser and Strauss, 1979; Strübing, 2004) and recommends immersing oneself in the data material at an early stage of content analysis in order to reveal its inherent meaning.

In preparation for the categorization, the complete transcribed text material was carefully read, important text passages were marked with the help of the QDA software MAXQDA and memos with comments and ideas for codes were prepared. In a first coding step, sense segments in the text were selected as coding units, to which different codes were assigned. In the spirit of “open coding” (Patton, 2015, p. 542) all text elements were included. In the text segments, this results in partial overlaps, i.e., different codes are assigned to a segment of meaning. A total of 673 codings were made. Some segments of meaning in the transcripts were assigned to several codes as the statements contain different aspects. The main coding of all text material was done by the first author herself. In addition, five persons (all with professional psychological-pedagogical knowledge) were available to evaluate the category system. They independently checked the fit on different text extracts and in some cases made their own suggestions for further codes. Based on the feedback from the co-coders and discussions, the category system was revised and supplemented.

In a further evaluation step, codes were grouped into superordinate categories. Common to the approaches of deductive and inductive categorization is that the text material is described and ordered on the basis of certain characteristics. These descriptive criteria are called categories in qualitative content analysis (Kuckartz, 2007, p. 57). According to Schreier (2014) the relevant aspects of meaning of the material form the categories of a category system. Some of these categories were derived from references to theoretical models and empirical research results, e.g., loss experiences or normative and non-normative life transitions in old age. Another part of categories was resulted from the questions of the interview guide. The latter categories include the burdens and problems as an example. In further steps, additional categories have been developed that relate to unexpected aspects or reflect developmental aspects of the individual partners and at the couple level during the counseling process (Flick, 2010).

## Central results

Because the results are very detailed and complex, we will first list the most important central findings in keywords and then explain them in detail:

- The reason for counseling was the aggravation of massive stress factors and experiences of loss, combined with subjective overstrain and despair, resulting in emotional alienation from the partner.
- Reciprocal emotional self-opening and change of perspective in the couples counseling process break through the dysfunctional patterns of couples.
- Emotional and physical rapprochement at the couple level, increased dissatisfaction with the couple relationship decreased and subjective wellbeing increased.
- Some respondents use avoidance or distancing for interpersonal problems in order to maintain harmony in the couple system and subjective wellbeing.
- Changes at the couple level after the end of couple counseling are not stable as soon as overwhelming stressors occurred again.

The last two sub-results suggest that communication about stressors may increase perceived stress, especially for partners who tend to use avoidant conflict resolution strategies; this could explain why increasing communication may well increase dissatisfaction in a partnership.

**Figure 2:**
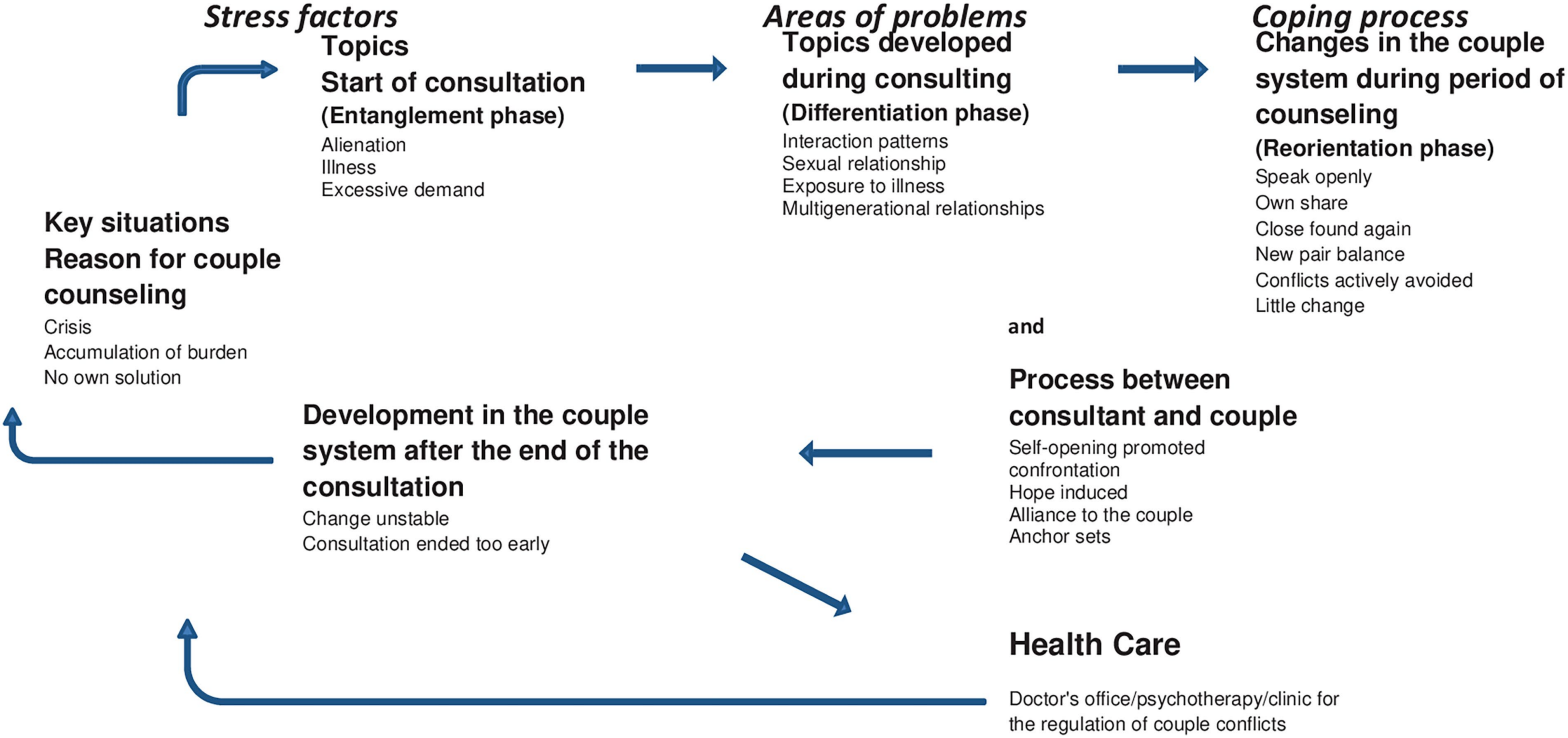
Long-term couple relationships-stress, problems and coping processes in couple counseling: A circular process model.

Respondents’ statements can be grouped under three broad thematic headings or clusters: Stress factors, problem areas and coping processes. These three thematic clusters follow, in the order given, the chronological sequence of the couple counseling processes as described in the interviews and are in a circular relationship. They are illustrated in Figure 2.

Stress-factors – Here, the bio-psycho-social limitations and couple-related difficulties are addressed that the interviewees experienced with regard to their long-term couple relationships. Burdening factors were found both in the occasions of registering for couple counseling and in connection with the topics at the beginning of the couple counseling process.

Problem areas – This category addresses the individual and couple-related aspects that the couples had to deal with and solve within the framework of couple counseling. In the course of the counseling process, the clients’ perspectives on the problem areas and dysfunctional interaction patterns within their couple relationship changed and expanded. The respondents reported that they developed a differentiated view of individual and couple- related aspects.

Coping processes – This category refers to the corresponding changing steps of the individual and the couple system. Various forms of coping with the burdensome factors and problems led to changes in the individuals and the couple system in the course of the couple counseling process.

The qualitative analysis of the interviews resulted in five main categories. The five main categories are named as follows: Key situations - reasons for couple counseling; Issues of the clients at the beginning of the couple counseling; Developing themes from clients’ responses during the couple counseling; Changes in the couple system during the counseling period; Development in the couple system at the end of counseling.

On the basis of the individual case analyses of the 10 interviews, it became apparent that it makes sense to categorize the interview statements along the time course of the couple counseling process, because the interviewees described a process with successive steps regarding couple counseling and the development of their couple relationship quality.

Similar to the three superordinate sections of the study, the phase model of systemic couple counseling according to Jellouschek and Kohaus-Jellouschek (1993) outlines ideal-typical chronological developmental steps that couples (can) go through in a joint therapy. These steps are the phases of entanglement, differentiation and reorientation.

At the beginning, the couple is in the so-called entanglement phase, which is perceived as a dead end.

In the study, this corresponds to the naming of the stress factors as reasons for couple counseling and the topics at the beginning of counseling. Table 1 gives an overview of the topics mentioned by the men and women at the beginning of counseling. Since this is a small sample, the percentages only serve as an overview for this small group and cannot be generalized.

**Table 1:** Overview of the topics mentioned by the men and women at the beginning of counseling. Percentage of mention of this problem for *N* = 10.

Categories	%
Emotional alienation of the couple	90
Massive health impairment	80
Despair/overstrain	80
Experience of loss	50
Problematic relationship with adult children	50

The interviewees did not see any possibilities of coping with these burdens and sought out couple counseling. The fact that the couples were confronted with their dysfunctional interaction patterns on the couple level in the counseling setting, and focused on their individual parts of the couple interaction pattern opens up the possibility for them to change into the so-called differentiation phase, in which they became aware of their entanglements on the couple level, exchanged information with their partner and subsequently came closer emotionally.

Characteristics of the differentiation phase can be found in the qualitative evaluation in the superordinate category “problem areas” which is classified as “topics developed by clients during couple counseling.” According to Jellouschek and Kohaus-Jellouschek (1993), couples succeed in solving destructive interactional cycles on the one hand by the partners individually (re)gaining more self-responsibility and on the other hand by the partners opening up to each other and showing their feelings. This brings movement into the solidified couple dynamics and the problematic issues can be worked on and overcome in the following “phase of restructuring” (or reorientation) in the context of couple counseling. In the main category “Changes in the couple system during the counseling period” there are corresponding cognitive, emotional, and behavioral aspects that the interviewees retrospectively named as relevant changes (Table 2). This phase can end with the couple either finding a new basis for the couple relationship or deciding to separate.

**Table 2:** Categories for which changes were reported during the consultation process.

Categories	*f*	%
Recognizing one’s own share	32	18.08
Talking openly with each other	29	16.38
Actively avoiding conflicts	26	14.69
Emotional closeness rediscovered	23	12.99
Change of perspective	19	10.73
Turning point	15	8.47
No change in the couple problem	14	7.91
Shared past as a resource	13	7.34
Physical rapprochement	6	3.39

In all 10 interviews, the respondents also spontaneously reported the development of satisfaction in their couple relationship after the end of the couple counseling. Satisfaction with couple relationships thus increased more or less markedly during the counseling period and decreased again after the end of the couple counseling.

Statements about the relationship with the counselor were also made by all respondents. The further seventh category is based on the statements made by the respondents regarding their contact with health care facilities. These two categories can be assigned to the category “coping processes.”

## Discussion

In this section, the central results of the study are presented and discussed in the systematics of the developed main categories, placed in a circular context (Figure 2)?

Both the results of the antecedent questionnaire study and those of the present study provide clues to the interlinking of the processes in the couple system with the emotions of the partners and with the state of health of the partners. Integrative theoretical models, which start at the interface of the areas of couple relationship, emotions and health, therefore provide a constructive framework for studies with long-term couples Uchino (2013). They show how specific components in couple relationships, for example basic social relationship processes and coordinated biological mechanisms interact in the partners and affect the individual state of health. In the process of emotion regulation, feelings, cognitions, evaluations, behaviors and (neuro)physiological components work together (Ochsner and Gross, 2007).

### Key situations/occasion

According to the subjective assessment of the interviewees, there had been problematic developments in the run-up of the counseling for quite some time. In older age, critical life events or human border situations are more likely to occur (Staudinger and Dittmann-Kohli, 1994). These include health restrictions, illnesses and experiences of loss of various kinds, which challenge the elderly to develop forms of coping and to comprehensively reconstruct their own life plan and personal sense system. At the time of application for couple counseling, the burdens in the couple system had either reached a “critical mass” and/or a current change in the couple and family system had occurred which gave the impetus to seek support through couple counseling (“key situation”). In this context, stressful events are to be understood as normative and non-normative critical life events (Filipp, 1990), e.g., chronic somatic and psychological illnesses (one’s own as well as those of the partner), the loss of one’s job or the exit from working life marked by unfavorable circumstances and subjective experiences of loss due to the departure of adult children. The stress factors were very heavy and powerful which in some cases persisted for years. The interviewees had previously tried in vain to cope with this negative stress without any external support which ultimately increased the strain. Attempts made by the individual, the couple and the help from their social environment had not brought relief and solutions therefore, professional help was then sought through couple counseling. This phenomenon is known as the cascade model (Bodenmann, 2000; Widmer Rodríguez Betancourt, 2001).

Studies have shown indications of the quality of relationships as a moderator for the physiological co-regulation of couples (Pietromonaco et al., 2013). Low levels of couple satisfaction increase the likelihood of developing a connection or transmission of negative moods and individual arousal between the partners. Couples who showed a high level of agreement in physiological arousal tended to be more likely to “infect” each other emotionally and to respond to stress and negative states of the other with their own arousal increase (Saxbe and Repetti, 2010). This is mediated *via* the HPA axis (hypothalamic–pituitary–adrenal cortex axis; McEwen, 2000). This important stress response system of the body is activated when stress is subjectively felt. It has cortisol as its end product and is considered a reliable predictor of stress. Results of two studies by Wrzus et al. (2013) support the hypotheses on overload and the connection with emotion regulation in older age. When older people are confronted with complex unpleasant events that affect several areas of their lives, they show more pronounced psychological and cardiovascular reactions than younger people.

In summary, it can be said that the key situations in which the couples chose external support through couple counseling were life situations in which the burdens and challenges of the couple’s relationship accumulated and the partners’ previous own coping capacities were no longer sufficient. These were the current worsening of a chronic conflict and/or a subjectively perceived crisis climax due to the accumulation of several prolonged burdens, in which the partners’ own coping abilities were exhausted. In some cases, additional acute stressful events occurred in the life context.

### Themes of clients at the beginning of couple counseling

The life situations that led the interviewed couples to turn to a counseling center include losses and limitations in the health sphere, i.e., somatic and psychological impairments and illnesses, as well as the loss of the job of one of them. At the same time, these are the issues that the interviewees recall in retrospect as their concerns in their couple life at the beginning of counseling. In the interviews, the spouses reported feelings of despair and being overwhelmed. In connection with this, a subjectively negative relationship quality increasingly developed in the run-up of the couple counseling. The topics at the beginning of counseling that were mentioned in the interviews largely correspond to the pre-concepts that can be found in the literature of the problem areas that people above 55 have: Experiences of loss, illnesses, insults and excessive demands as well as transitional situations such as retirement or the so-called “empty-nest situation” (Freund et al., 2014; Riehl-Emde, 2014, 2015). Massive health impairments due to physical and mental illnesses and symptoms were the most frequently mentioned category at the beginning of the couple counseling process. These were, for example, chronic somatic and psychological illnesses and limitations of one or both partners, which are sometimes accompanied by permanent pain, physical and long-term psychological consequences of a serious somatic illness, depression, anxiety disorders or an addiction of one partner. Experiences of loss were also frequently mentioned, especially the loss of a job or bullying experiences, but also the departure of the adult children and thus the farewell to the active role as parents. It became clear that these were cumulative experiences of loss, i.e., successive or simultaneously occurring stresses that exceeded the coping possibilities of the individual partners and the couples in the social environment. Situations of overstrain and feelings of despair were described: “And it went on like that for 5 years and then our family doctor - I complained again and again, and then he said ‘Yes, yes, Mrs. A., you must be glad that your husband is still alive at all!’ I say, yes, of course I’m glad he’s alive, I say, but that’s not life any more. I say I’m more likely to die from it than he is.”

These stresses and experiences of excessive demands were accompanied by “emotional alienation” at the couple level: “So these were concentrated situations that came in our way and we grew more and more apart. He was always in his space and I was in my space.” This category captures the couple’s lack or loss of emotional contact. “He was always in such a glass bell,” is how one respondent describes her impression of her husband. Experience of emotional alienation from the partner is taken up in stress research in connection with the effects of stressful situations on close relationships (Karney et al., 2005).

The approach of developmental psychology of the lifespan (Brandtstädter and Lindenberger, 2007) offers a suitable theoretical framework for considering the self-regulatory processes and abilities of individuals as well as dyadic processes in the design of the long-term couple relationship in older age. Research results on action motivation and emotion regulation in older people in long-term couple relationships are mainly relevant here (Carstensen and Lang, 2007; Charles and Carstensen, 2007). With decreasing remaining life time, emotion-related goals are prioritized (Carstensen et al., 1999, 2011), i.e., the quality of social relationships - especially the couple relationship - becomes more significant in older ages. The basic need for closeness and acceptance in the couple relationship is thus given a special importance. If the bond with the partner appears to be at risk, it can trigger considerable stress. The close relationship with the partner therefore influences health by changing the health-relevant physical reactions of the partners in the process of couple interaction, which arise as correlates of their emotions (Sbarra and Coan, 2018).

At the beginning of counseling, the partners described themselves as being emotionally distant from each other. “Emotional alienation” in the sense of loss of intimacy and access to the partner combined with feelings of isolation were consistently mentioned as a phenomenon by the interviewees: “Everyone had lived his or her own life… we had not lived as a couple at all over the years.”

This experience of emotional alienation from the partner is taken up in stress research in connection with the effects of stressful situations on close relationships (Karney et al., 2005). Neff and Karney (2004) studied the “stress spillover” effect. According to this, couple-external stress causes negative perceptions to occur in the couple relationship and the partners’ constructive handling of these critical perceptions is severely limited. When stress is high, the partners’ cognitive resources are overtaxed and they cannot differentiate between external stress and relationship dissatisfaction. One interviewee described her situation as follows: “… I soon had a nervous breakdown. I screamed in the middle of the night that the whole house must have heard everything. From despair and not being able to do it anymore.”

According to Bodenmann (2013), a close, sustainable and binding couple relationship fulfills the basic human need for attachment. The threat of losing the couple bond can trigger anxiety. Mentalization-based approaches in couple therapy indicate that in stressful and threatening situations in which fear of loss of attachment is experienced, the attachment system undergoes considerable activation. In threatening situations, the partner-related attachment has the adaptive advantage that the proximity of the partner secures support and helps to reduce fear (Rohmann et al., 2006). In parallel, the partners’ ability to mentalize may be (temporarily) limited. One of the interviewees described her spouse as follows: “He was actually always in a glass bell jar. So, you could not get close to him at all.” This leads to misinterpretations and dysfunctional reactions in the couple interaction, which in turn activates anger and withdrawal reactions in the other (Plitt, 2017). Here, the concept of mentalization seems to be relevant. The concept of mentalization goes back to the working group around Fonagy (Fonagy et al., 2002) and includes “the ability to appropriately imagine inner states such as motives, feelings and beliefs in one’s own self as well as in other people” (Rottländer, 2017, p.24). However, mature mentalizing skills promote joint problem solving as a couple. Mentalization is conceived as a stress-dependent process (Kirsch et al., 2016). Especially in the case of strong emotions, e.g., fear or anger, which are accompanied by high arousal in the partners, the risk is high that–temporarily–the ability to mentalize appropriately cannot be maintained. According to the dual stress processing model (Luyten et al., 2011), arousal increases under stress and leads to a switch from cortical (prefrontal cortex) to subcortical systems (posterior cortex and limbic system) (Schultz-Venrath, 2015). For reflective problem solving with low to medium arousal, cortical systems are needed. With high arousal, subcortical services enable rapid information processing, but these are associated with a decline in mentalizing ability. Explicit, controlled mentalization processes decrease with increasing arousal. Implicit, automatic mentalization processes take their place. In the neurobiological stress-dependent model, the individual switching point from an explicit to an implicit mentalization mode is related to the extent of attachment stress and individual coping strategies (Kotte and Taubner, 2017). Stress and attachment activation in the couple relationship is closely linked, because the switching point from explicit to implicit mentalization is related, among other things, to the extent of attachment stress and the individual coping strategies. A sufficient mentalizing ability promotes constructive handling of one’s own emotions and those of the partner and makes it possible to regulate the central conflicts in the couple relationship.

In summary, the issues that the respondents were aware of at the beginning of counseling were massive health burdens and various experiences of loss. This was associated with feelings of being overwhelmed in both partners and, as a consequence, emotional alienation as a couple. In the case of over-demanding stress experiences, mature forms of mentalization competence may break down in the partners. This leads to misperceptions and dysfunctional experiences in the couple relationship (Bark et al., 2016). The attachment relationship of the partners was in danger at the time of enrolment in couples counseling which due to the high priority of emotionally significant relationships among older people, means that emotional security in the couple relationship was no longer available to them as a resource.

### Themes developed by clients during the counseling process

This category differs from the category “Themes of the clients at the beginning of the couple counseling” in that during the course of the counseling process the clients took a closer look at certain aspects or background themes of their strained couple relationship and their own share in the problems and opened up to them. This changed the couples’ perspectives. Instead of the topics at the beginning of the couple counseling, which they had described more in terms of external stress factors, the focus was on internal concerns and needs of the interviewees. This change of focus can possibly already be seen as a result of the couples counseling. In current couples therapy research, it is assumed that the approaches whose effectiveness has been proven evidence-based have common factors of action (Davis et al., 2012). Thus, non-specific mechanisms of change are identified in couples therapy, which can be summarized in three categories: “changing the doing, changing the thinking, and changing the feeling” (Davis et al., 2012, p.42).

The topics of the interviewees largely coincide with the topics listed in the literature as counseling themes of older couples in couples therapy (Riehl-Emde, 2014, p.15). The interviewees described interactional dysfunctional patterns within the couple relationship that had developed, for example, as a consequence of the changed situation following the termination of employment or due to an illness: “… and, that was clear to us relatively quickly that this is already a big point with us that is simply not okay. Because everyone says, listen, I cannot do this and that because the other person is not well. From both sides, this consideration was just too much of a thing.”

The relationship with the adult children and the empty-nest situation as well as their effects on the quality of the couple relationship became relevant themes for the interviewees in the course of couple counseling. Relationships with family members, especially ties with one’s own children and grandchildren, are an essential source of quality of life and wellbeing throughout the life cycle (Mahne and Huxhold, 2017). Adult children who lived in the parental household until recently had a regulative function in the couple system. For example, they were confidants of a partner and compensated for emotional deficits in the couple relationship: “It was positive, I could always talk to her (the daughter, author’s note), which I could not do with my husband.” This compensation possibility had been lost in the life situation of the interviewees before the start of counseling, so that the couples were challenged to find new regulation mechanisms for distance and closeness in their contact.

In their role as parents, the couples were concerned with the lifestyle of their adult children and were emotionally engaged in this respect: “And that’s how I am again now. I try, the negative is there, the worry about the daughter is there, in order not to get broken by it, because the doctor himself already said when she gave us, when she once made such a scene to me, that I had not taken care of her, with problems in her marriage.”

The imbalance in the couple relationship due to illness or the biographical burden of a partner was also addressed in the counseling process. For example, it was about the unequal distribution of responsibility: “That goes too far. Then I also say, why do not you do it this way, and why do not you do it that way. Because then I’m already somewhere in this stupid feeling of responsibility and then of course I overshoot the target…” Maladaptive strategies of the partner toward the patient - e.g. overprotection - cause one’s own negative stress level to increase (Coyne and Fiske, 1992).

The organization of physical contact between the partners and the disruption of their sexual relationship was related to health problems, emotional alienation from each other or power struggles within the couple relationship. In a Canadian longitudinal study of nearly 400 elderly couples living at home on their own, common sexuality was predominantly positively related to couple quality and seen as a source of satisfaction (Trudel et al., 2014). According to the interviewees, sexuality was only addressed in some couple counseling processes. In the interviews almost all respondents addressed the - missing – physical contacts, tenderness with each other and the sexual relationship as a couple. The topics of “distance and closeness” and “attachment and autonomy” within the couple relationship played a role. Interaction behavior of a partner that was perceived as possessive or emotionally distant was sometimes answered by the fact that the other partner avoided sexual contact: “And then you really noticed, no, that: ‘If I kiss now, then maybe he’ll get me’. No! That’s when she called it a day. As if she was closing down. She’s pissed now. The basic sourness, I would say, prevents her from letting go and saying, ‘Oh, come on!’” In one couple, both interviewees explicitly spoke of “punishing” the other by refusing to engage in sexual activity. There are many indications that satisfaction with the sexual relationship is not mainly determined by whether sexual intercourse takes place, but whether a couple is able to exchange information about their individual wishes and needs and to agree on the design of their common sexuality (Baas and Schmitt, 2010).

In addition to the stressful aspects, the respondents explicitly named resources of their couple relationship. Mutual solidarity, continuity in the bond with the partner and common values were evaluated as the basis of the couple relationship and as stabilizing factors. Here, the focus was on the long duration of the couple relationship and the value of “staying together” due to the common life story. “We have always been a couple. And I do not know, I do not give up so easily either. And neither does my husband, I guess. Our marriage, our family actually has a very different basis or goal than the young people of today.” In the concept of commitment, which includes the conscious decision to bind to the partner and continuity over time, similar characteristics can be found (Sternberg, 1986; Acker and Davis, 1992; Bodenmann, 2013).

In summary, it can be said that in the course of the counseling process, the respondents became aware of their dysfunctional interactional cycles as a couple and their own contribution to these patterns in their couple relationship. In this respect, the differentiation of the topics for couple counseling, taking into account dysfunctional patterns in couple interaction, is already a significant mentalizing achievement that contributes to the couple’s ability to solve problems (Plitt, 2017). An imbalance in the distribution of roles due to illness, restriction or biographically determined vulnerability of one partner was addressed as well as the upcoming developmental tasks in the relationship with the adult children in the sense of a detachment from the joint parental role. Discrepancies in the couple’s physical/sexual relationship came up in some of couples counseling processes. Addressing the partnership problems at the beginning of the couple counseling process caused both partners to develop a greater awareness of the tensions and unresolved issues in the couple relationship.

### Changes in the couple system during the counseling period

Changes are understood here as coping processes of the partners. The changes described in the interviews concerned the couple relationship and the interviewees’ *per se*. These changes took place within and outside the couple counseling sessions. The following experiences in the couple’s interaction process within the counseling sessions were relevant for the interviewees.

The open exchange about their thoughts, feelings, experiences, problems and perspectives in relation to their partner was mentioned very often: “One has, one has been more open with oneself. They talked more about themselves personally. You also said your own wishes in public sometimes. And that went down very well.” One woman interviewed emphasized that in the counseling situation she was able to listen to her husband and take in his impulses: “And then I say, you hurt me even more when I sit there now and suddenly hear something from you that I could have tolerated before, but did not hear. And that astonished me, made me a little angry. But I never got up and went out, because somewhere, somewhere it was perhaps also interesting.”

Studies on general effective factors in couple therapy identify the following overarching therapeutic processes: changing the couple’s view of the named problem toward objectivity, contextual involvement at couple level, reducing emotion-driven dysfunctional behavior, promoting previously avoided expression of emotions and constructive communication patterns (Benson et al., 2012). The degree of openness and relatedness to the partner may influence the effect of stress on the HPA axis (Slatcher et al., 2010).

Recognizing one’s own part in couple’s problems was a decisive step for the interviewees: “No, actually just as, uh, insecure or that I know that ultimately I have to take the steps. Um - yes, and I’m just too cowardly to say, okay, it’s not the way we imagine a marriage to be. And, uh, yes, no one can change inwardly like that, that’s not possible at all…” Inspired by the counseling, more than half of the interviewees changed their perspective on the couple relationship: “Well, by trying to approach the man, my partner, and trying to see it through different eyes.” Research on couple therapy shows that therapeutic processes that initiate changes at the emotional, cognitive and behavioral levels in parallel, prove effective (Greenberg and Johnson, 1986; Christensen, 1988; Greenberg et al., 2010). In the mentalization concept, perceiving, classifying and communicating one’s own affects is seen as an achievement, as is perceiving the emotions of the partner (Cordes and Schultz-Venrath, 2015). This enables them to adopt the perspective of the other person and to see the effect of their own behavior from the partner’s point of view. The interviewees described this change of perspective as a decisive step in the process of couple counseling.

The respondents described that in the course of counseling they realized that the dysfunctional interaction patterns at the couple level had led them to emotional alienation and that they had lost emotional access to each other in permanently overstressing situations: “The wall, it went up.” The threat to attachment in the couple relationship is highly linked to stress (Hazan and Shaver, 1987). This is consistent with the model of stress-dependent switching of mentalization (Luyten et al., 2011). Stress, heightened arousal and the accompanying activation of the attachment system result in a switch from explicit controlled to implicit automatic mentalizing (Plitt, 2017, p.44). Depending on individual attachment experiences and psychological structural characteristics, this switch occurs at different times. In cases of high emotional stress, as described by the respondents, partners may not have had access to their mentalizing abilities. The model of coregulation in close relationships assumes that the partners in couple relationships form a dynamic emotional system (Butler and Randall, 2013). During the couple counseling sessions, the respondents were able to lower their arousal and were (again) able to mentalize each other and reflect on their couple dynamics.

The respondents described changes outside the counseling situation, i.e., in the couples’ everyday life. They had regained emotional and sexual/physical closeness to their partner, which also increased their subjective wellbeing: “Well, where - in the beginning, where we talked about it a lot, I thought that was great for both of us! He also became more and more open, and that was very, very nice. It made me feel better and better, and I also noticed how my body reacted differently. And the aches and pains that you have now became less and less.”

Primary processes of change within the counseling sessions, which enable couples to experience intimacy in the short term, are distinguished from secondary processes of change outside the therapeutic situation (Schär, 2010). The experience of intimacy with the partner within the couple therapy session, supported by therapeutic interventions, promotes the transfer into the couple’s everyday life. The respondents reported that shortly after the couples counseling sessions they implemented changes in their joint interaction as a couple in everyday life. In the model of SAVI (Strength and Vulnerability Integration), age-related socio-emotional strengths are postulated, which are countered by age-related vulnerabilities that manifest themselves in situations in which persistent emotional arousal occurs, which can only be reduced with a time delay in older age (Charles, 2010; Charles and Luong, 2013). A new balance as a couple was found, which one of the interviewed women expressed as follows: “And, but it’s like this - 50 years, soon 50 years we have been together, it’s like here, a chain link, sometimes it’s messed up, the link, and then it’s smooth again, or you pull it smooth. And it’s the same in marriage. Sometimes there are times that are so tangled up that you think you will not get them straight again, and then they become smooth again.” The connection between physical and psychological wellbeing and satisfaction in the couple relationship has been proven many times and is probably influenced by such psychobiological stress reduction processes (Frisch et al., 2017).

Some respondents tried to stabilize the couple relationship by practicing active avoidance of confrontational encounters with the partner on long-known conflict issues. “How do I say this? How did I do that? When I use that now, the word, submissive I gave myself. … So that she got the upper hand again - that is, now we have 4 days again, when she has been here, she was unresponsive for 3 days again.” A conscious decision of being considerate or “willing to sacrifice” served some partners to take the negative tension out of the current couple interaction. “Yeah, and now we have sort of settled there in the middle. Um, I try not to provoke her, and she does not always take everything so badly. That’s an advantage.”

In long-term couple relationships in older ages, maintaining the level of functioning in the couple relationship is experienced as a gain, as it promotes subjective wellbeing (Ebner et al., 2006). According to Gross’ process theory (John and Gross, 2004), older people more often choose the affect regulation strategy of reappraisal, in which they reinterpret the connoted or stressful situations. In this way, they avoid negative emotions and cope with the situations by using fewer resources. The use of passive emotion regulation strategies is part of the expanded socio-emotional competencies in older ages (Blanchard-Fields, 2007). Constructive and relationship-promoting conflict avoidance strategies are thus more frequently found in couple conflicts among the older people than in younger people (Holley et al., 2013). In older people, avoidance goals that are directed against a loss or a negative change of the currently stable status can thus be evaluated as constructive (Urry and Gross, 2010). Strategies of repression or suppression in emotionally significant conflicts in couple relationships require considerable cognitive resources (Edelstein and Gillath, 2008; Peters, 2015a). It must therefore be asked under which conditions the described avoidance strategies are to be regarded as a mature form of affect regulation, and when they reach their limits. This can occur when the physiological arousal reaches such a high level that a situation of excessive demands arises due to the age-related prolonged arousal (McEwen, 2000; Labouvie-Vief and Medler, 2002; Charles, 2010).

For the interviewees, making a new decision for their partner meant a far-reaching change, which was also described as a turning point: “I still said, here you are again at last. Yes, and such a small occasion, big effect, can make you realize, like brooding and pondering 2 days before, can make you realize how valuable the person actually still is.” The long history as a couple was brought into view as a resource. Affect optimization theories (Carstensen et al., 2003; Charles, 2010) postulate that so-called good aging is characterized by self-effectively shaped positive present. Current approaches to affect reactivity (Ong and Bergeman, 2004; Almeida, 2005) emphasize that in stressful situations the extent of negative affect tis not that significant, but rather the possibility of experiencing positive emotions. The fact that they remembered good times in the couple relationship despite the current stresses and difficulties made it easier for the older adults to regulate their emotions (Riehl-Emde, 2016a,b).

There were clear indications from some respondents that they had not perceived any change in their couple issues. Four respondents from two of the couples reported that they had changed the way they dealt with an unbridgeable area of conflict. An increase in intimacy inside couples is supported by therapeutic interventions that promote the following factors in the partners: Empathy, insight into motives and feelings, insight and behavior change, and mobilization of support (Woolley et al., 2000).

In summary, it became clear that in the course of couples counseling relevant intra- and inter-individual changes occurred in the clients and in their couple relationship. Addressing the problems in the partnership brought about a greater awareness of the dysfunctional interaction circles and unresolved issues in the couple relationship. Through self-critical reflection of their own parts in these interaction patterns and emotional self- opening toward their partner, the respondents broke through their previous way of shaping the relationship. This enabled the couples to get emotionally and physically closer again. According to Riehl-Emde (2005), sexuality in old age contributes to people feeling secure in the couple relationship and close to their partner, which in turn supports the ability to regulate self-esteem. Tenderness is now considered an underestimated factor in the state of health in the third and fourth ages (Riehl-Emde, 2016a). For some of the respondents, “active avoidance of conflict” served to regulate negative emotions in the couple relationship.

### Development in the couple system after end of counseling

The respondents retrospectively described that they experienced an emotional rapprochement with their partner during the course of the couple counseling process and implemented positive changes in couple communication as well as changes in their interactional patterns as a couple. This led to improved subjective wellbeing. However, the changes tended to be short-lived. Toward the end or after the end of the couple counseling process, the subjectively perceived stress and dissatisfaction regarding the couple relationship increased again for the majority of the respondents. This development can be clearly seen in the assessments of the quality of the couple relationship, which the respondents made during the interviews in a coordinated system (Figure 1). After an increase in the first phase of couple counseling, couple satisfaction dropped again more or less. It is very clear from the interviewees’ statements that they often experienced the changes achieved during the counseling as not stable: “First momentum, then the old rut.” The interviewees did not succeed in transferring the changes to everyday life in the long run. This was especially the case when negative stress factors reappeared: “Let us not talk much to each other because we both cannot anymore. And we actually wanted to get rid of that, … And that’s not where we are at the moment. And that’s why it’s still incredibly difficult for us, for me, at the moment.”

In the synopsis of all 10 assessments, it becomes clear that after an initial increase, satisfaction with the couple relationship drops again (Figure 1).

The interviewees said that they had ended the couple counseling too early or would seek counseling again in the event of further critical situations: “We also said afterwards ‘We should not have given up so soon! We should have come here longer, talked longer and more, maybe it would have been even better. Maybe we broke off too soon.” We said, ‘OK, we’ll manage. We’re on the right track, we’ll get there’. But it was still a long way from being really solidified.” With increasing attachment stress, which those we interviewed experienced as stressful, a loss of emotional contact at the couple level and a decrease in the flexibility of the partners in reflecting can be assumed. Situations with high emotional arousal–such as stressful conflicts in the couple relationship–are associated with increasing physiological activity in older people (Kunzmann and Grühn, 2005; Uchino et al., 2005). Older people, unlike middle-aged adults, are thought to show less physiological reactivity when their arousal is low. If the emotional arousal caused by socio-emotional stress exceeds a threshold, older people show a stronger physiological reaction than younger people and as a result cannot use their crystalline intelligence skills to solve problems (*cf.* Peters, 2015b).

Within the framework of an integrative couple therapy model (Schär, 2010), a distinction is made between primary and secondary processes of change. In the short term, at the beginning of couple therapy, it is promoted that the couple experiences intimacy with each other (again) in different areas of their relationship. On this basis, interventions can be used at a later stage in couple therapy to promote the couple’s experience of intimacy in the long run and subsequently couple satisfaction and couple stability.

In summary, it can be said that the majority of the interviewees found emotional and physical closeness in their couple relationship again during the couple counseling, perceived positive aspects in their partner and in the relationship and ended the couple counseling at this point. However, these aspects of change did not prove to be stable when intense stress occurred again. Conflicts on the couple level then increased again and both partners showed aggressive behavior as a result of their renewed overload. As one interviewee said: “Because simply both partners are physically in such a bad state and then only, not with each other, but only against each other. Everyone is exhausted, annoyed, but wants to finish their work.” Due to the increased stress level and the activation of the attachment system, the explicit mentalization ability of the partners could have been limited. Cognitive capacity is lower due to switching to the implicit mentalization mode at high stress, which makes memory performance, information processing and problem solving more difficult. Higher emotional arousal due to chronic stress is coupled with a lower memory of learned communication skills in couples (Baucom et al., 2012).

The interviewees stated that they should have continued or resumed couple counseling for a longer period of time. Problems in the couple relationship were named in the couple counseling, but the couples did not (yet) find viable solutions.

### Use of health system facilities to regulate couples’ conflicts

In addition to the three central themes “stress factors, problem areas and coping processes,” which can already be found in the guideline for the interviews, it came up again and again in the interviews surprisingly that the interviewees made use of facilities of the health system, parallel to the counseling process or before and/or after it when the tensions in their couple relationship and consequently the subjective stress increased strongly. They made contacts with a general practitioner or a specialist, with psychotherapists in private practice, with a specialist psychiatric clinic or with a general hospital.

Massive acute and chronic health impairments–due to both physical and mental illnesses–were among the central stress factors that the interviewees mentioned as characteristics of their life situation. It was therefore to be expected that they would also seek help from the health system during the couple counseling process and/or afterwards. In some cases, however, the interviewees explicitly stated that their partner’s stay in an inpatient facility or medical treatment after the end of couple counseling served to regulate the emotions in the couple relationship. With regard to her husband’s inpatient stay in a psychiatric hospital, one woman said: “It is not a matter of deporting him and taking him away. But simply so that I can get some air. No, I do not want to leave or anything. I just want, we talked on the phone every evening, but I just want to have the feeling that he’s okay for now. They’ll build him up again, and then I do not need to do that. So according to the motto, I can look after myself a bit and do not have to - that’s actually how it is, I’ve already told him so often, I also said that here at the time, I just do not want to have the responsibility.”

In some cases, stress in the couple relationship had contributed to symptoms on the physical level that resulted in getting a medical treatment. One of the interviewees reflected: “Yes, and whether she told him that, I do not know, in any case there was the examination (at the family doctor’s, author’s note) and was able to put him in hospital, and that was more or less, I would almost like to say, that was the salvation of our marriage.”

After hearing such statements in the interviews, questions arise about the networking of psychological counseling center and the health system. Effectiveness studies on family and couple therapy treatments indicate that medical treatments are less used in parallel. In particular, this is to be observed in so-called “high-utilizers” (Crane, 2007). For the majority of the interviewees, the initial indication to turn to a counseling center for couples counseling came from a medical or psychotherapeutic practice. The criteria of such referrals to couple counseling were not known in the context of this study. Neither was it known whether and how the services and interventions in health care facilities and in couple counseling were coordinated with each other. The question arises whether couples seeking counseling would make less use of the health system if the problems at the couple level were eased through couple counseling. Research on the relationship between frequent use of health care facilities and psychotherapy or family and couple therapy shows an “offset effect.” Among those who used marriage or family therapy, the use of the health care system decreased by over 20% (Law and Crane, 2000).

The interviewees described that they used facilities of the health system to regulate their emotions when interpersonal situations in their couple relationship escalated. Based on these statements, it can be asked whether and how the networking of counseling center with the health system takes place. What criteria are used to refer couples to counseling center and then from these centers to health care facilities? Can the services and interventions in the counseling and health sectors be coordinated more effectively to meet the needs of those seeking advice? In order to maintain their subjective wellbeing, it may be advisable for older people to make use of facilities in the counseling and health sectors in parallel. The interventions of different help and therapy services should be coordinated as much as possible in order to achieve synergy effects. This would require cross-case and case-related contacts between the facilities.

### Limitations of the study

Overall, the generalizability of the results of the study is severely limited due to the data basis. It was not possible to select the participants in the qualitative interviews according to certain criteria. It is questionable whether clients who were more satisfied with the couple counseling process and with the counsellor agreed to participate in the interviews. Couples who had separated or dropped out of counseling, for example, were not available for interviews.

The validity of the data is also limited by the characteristics of the sample. They were heterosexual couples aged 55 and over who had been married for many years in their first marriage. According to the counselors, the couple counseling ended normally and the couples were invited by their respective counsellor to participate in the questionnaire survey and in an interview. It is possible that any negative critical aspects of couple counseling were not mentioned in the interviews. The fact that the partners knew that the other person would also be interviewed on the same topic could also have led to the respondents’ selective statements.

## Conclusion and outlook

Long-standing couples seek couple counseling when they experience excessive stress due to the accumulation of chronic and acute stresses and limitations associated with the subjective feeling of being overwhelmed and helpless. When they register for couples counseling, they experience very high emotional arousal due to being inundated with problems and finding no coping options of their own.

Due to the increased stress level, the partners lose access to their socio-emotional expertise that were acquired in the course of their lives and their problem-solving abilities in the couple relationship.

As a result of the overload, there is emotional alienation at the couple level, which activates the couples’ attachment system. As a result, the partners get into further emotional turmoil.

The partners’ ability to mentalize temporarily breaks down under their high stress load, which leads to a disruption of the couple’s affective communication and sometimes to aggressive behavior of the partners. Individual vulnerabilities and attachment styles influence the switching point from explicit to implicit mentalizing in the couple system.

The counseling process enables the partners to emotionally re-approach each other on the couple level, which in turn leads to the emotional calming of the individuals. The couples resume physical touch, which calms the bonding system of the partners and reduces negative emotional arousal.

This calming down enables the older couples to access their acquired socio-emotional expertise again. By reducing arousal, the partners are (again) able to open up emotionally to each other, to take the other’s perspective and to grasp their own part in the problems at the couple level (i.e., to use mature mentalizing). This enables reflective problem solving at the couple level, and the partners see in a differentiated was problem areas as well as resources in the couple relationship.

In the case of long-known or existing interactional problems in the couple relationship, older people use situational and constructive avoidance and distancing strategies that help them to avoid emotional stress in the couple relationship and to stabilize their emotional well- being.

If significant stress occurs again, the likelihood increases that older adults are not able to avoid high levels of distress or to use cognitive-behavioral strategies to mitigate aversive emotional experiences. As a result, their mature mentalizing capacity breaks down again. In these situations, they can only access their age-related emotional advantages in order to regulate their emotions to a limited extent.

Some older people in long-term couple relationships use health care facilities in stressful situations to get support in emotion regulation and to bring about relief in their couple system.

Even though the questionnaire study preceding the present study presented here is not central to this paper, two of the results are noteworthy: We found a decrease in subjective complaints and overall burden on the couple relationship, but at the same time an increase in dissatisfaction with affective communication/emotional intimacy, problem solving and aggression in the couple relationship. This last result can be explained by partial results of the study.

These assumptions form the basis for further research questions in the counseling of long-term couples. In this context, knowledge about the changes in emotional regulation strategies with increasing age remains of particular importance.

### Practical implications for consulting with long-term couples

From our point of view, the results lead to assumptions about the design of the counseling process with long-term couples, which we have written down here as summarizing thoughts:

- Consider acute and chronic psychosocial stressors and resources as contextual factors and extent of fragilization.
- Consider results from affective neuroscience (arousal and coregulation processes in couple relationships).
- Focus on the age-dependent priority of emotional security and connectedness in the couple relationship as a basis for the constructive handling of conflicts and wellbeing.
- Mature strategies of emotion regulation, e.g., active avoidance of destructive conflicts in the case of “unsolvable problems” and promote interpersonal competences (de-escalation, self- opening, change of perspective, own assumption of responsibility).
- Targeted cooperation of couples counseling/therapy with institutions of the health system.
- Offer catassignmental consultation appointment for stabilization, encourage re-reporting in case of renewed stress.

## Data availability statement

The raw data supporting the conclusions of this article will be made available by the authors, without undue reservation.

## Ethics statement

Ethical review and approval was not required for the study on human participants in accordance with the local legislation and institutional requirements. The patients/participants provided their written informed consent to participate in this study.

## Author contributions

All authors listed have made a substantial, direct, and intellectual contribution to the work and approved it for publication.

## Conflict of interest

The authors declare that the research was conducted in the absence of any commercial or financial relationships that could be construed as a potential conflict of interest.

## Publisher’s note

All claims expressed in this article are solely those of the authors and do not necessarily represent those of their affiliated organizations, or those of the publisher, the editors and the reviewers. Any product that may be evaluated in this article, or claim that may be made by its manufacturer, is not guaranteed or endorsed by the publisher.
